# High *FAAP24* expression reveals poor prognosis and an immunosuppressive microenvironment shaping in AML

**DOI:** 10.1186/s12935-023-02937-3

**Published:** 2023-06-17

**Authors:** Xiebing Bao, Jingyun Chi, Yiwei Zhu, Minfeng Yang, Jiahui Du, Zaixiang Tang, Xiaogang Xu, Genxiang Mao, Zhibing Wu, Jun Chen, Jingsheng Hua, Ting Xu, Song-Bai Liu

**Affiliations:** 1 National Clinical Research Center for Hematologic Diseases, Jiangsu Institute of Hematology, The First Affiliated Hospital of Soochow University, Suzhou, 215006 China; 2 Suzhou Key Laboratory of Medical Biotechnology, Suzhou Vocational Health College, 28 Kehua Road, Suzhou, 215009 China; 3 Institute of Blood and Marrow Transplantation, Collaborative Innovation Center of Hematology, Soochow University, Suzhou, 215006 China; 4 Suzhou Medical College, Soochow University, Suzhou, 215006 China; 5 Department of Health Technology and Informatics, The Hong Kong Polytechnic University, Kowloon, Hong Kong SAR P.R. China; 6 Department of Biostatistics, School of Public Health, Medical College of Soochow University, Suzhou, 215123 China; 7 Zhejiang Provincial Key Lab of Geriatrics & Geriatrics Institute of Zhejiang Province, Affiliated Zhejiang Hospital, Zhejiang University School of Medicine, Zhejiang University, Hangzhou, 310058 China; 8 Department of Hematology, Taizhou Municipal Hospital Affiliated to Taizhou University, Taizhou, 318000 China; 9 Department of Oncology, Affiliated Zhejiang Hospital, Zhejiang University School of Medicine, Hangzhou, 310013 China

**Keywords:** *FAAP24*, AML, Prognosis, Immunosuppression, Chelerythrine

## Abstract

**Background:**

As a core member of the FA complex, in the Fanconi anemia pathway, *FAAP24* plays an important role in DNA damage repair. However, the association between *FAAP24* and patient prognosis in AML and immune infiltration remains unclear. The purpose of this study was to explore its expression characteristics, immune infiltration pattern, prognostic value and biological function using TCGA-AML and to verify it in the Beat AML cohort.

**Methods:**

In this study, we examined the expression and prognostic value of *FAAP24* across cancers using data from TCGA, TARGET, GTEx, and GEPIA2. To further investigate the prognosis in AML, development and validation of a nomogram containing *FAAP24* were performed. GO/KEGG, ssGSEA, GSVA and xCell were utilized to explore the functional enrichment and immunological features of *FAAP24* in AML. Drug sensitivity analysis used data from the CellMiner website, and the results were confirmed in vitro.

**Results:**

Integrated analysis of the TCGA, TARGET and GTEx databases showed that *FAAP24* is upregulated in AML; meanwhile, high *FAAP24* expression was associated with poor prognosis according to GEPIA2. Gene set enrichment analysis revealed that *FAAP24* is implicated in pathways involved in DNA damage repair, the cell cycle and cancer. Components of the immune microenvironment using xCell indicate that *FAAP24* shapes an immunosuppressive tumor microenvironment (TME) in AML, which helps to promote AML progression. Drug sensitivity analysis showed a significant correlation between high *FAAP24* expression and chelerythrine resistance. In conclusion, *FAAP24* could serve as a novel prognostic biomarker and play an immunomodulatory role in AML.

**Conclusions:**

In summary, *FAAP24* is a promising prognostic biomarker in AML that requires further exploration and confirmation.

**Supplementary Information:**

The online version contains supplementary material available at 10.1186/s12935-023-02937-3.

## Introduction

AML is a heterogeneous hematological malignancy caused by malignant transformation of hematopoietic stem cells that is characterized by an increase in clonal myeloid primordial cells [1]. AML patients have a very variable prognosis and a high mortality rate: the 5-year survival rate is approximately 27% for people older than 20 years and approximately 69% for people younger than 20 years [2]. The pathogenesis and mechanism of AML are extremely complex and mainly include environmental factors and genetic factors.

Genetic mutations in cell proliferation, differentiation and apoptosis pathways are the basis for the pathogenesis of AML [3, 4]. Mutation also results in abnormal gene expression of oncogenes or suppressor genes. For example, mutation of the transcriptional regulatory *factor TP53* upregulates the expression of genes in cancers [5, 6]. With the advent of personalized medicine, many diagnostic and prognostic biomarkers with disordered expression levels have been identified and contribute to a better understanding of the molecular pathology of the disease [7].

Forming a heterodimer complex with its partner *FANCM*, *FAAP24* (FA-associated protein 24) can recognize and bind to damaged DNA and recruit other members of the FA pathway to initiate downstream repair [8]. Deficiency of *FAAP24* causes a cancer-prone recessive genetic disorder characterized by congenital abnormalities, bone marrow failure, and cancer susceptibility [9]. According to structural analysis, *FAAP24* has a high affinity with single-stranded DNA (ssDNA), which is required for optimized checkpoint activation. Its ssDNA-binding activity and FANCM-interacting functions enable *FAAP24* to respond to DNA damage against crosslinking lesions [10]. The Fanconi anemia DNA repair pathway, in which *FAAP24*  involves, has recently been intensively studied for its contribution to anticancer drug resistance [11]. Therefore, functional research on *FAAP24* has also been extended to other cancers, including human clone cancer [12]. However, its prognosis-impacting function in AML is rarely discussed.

Through an analysis of the public databases, we investigated *FAAP24* expression differences across cancer types. We evaluated the independent prognostic value of *FAAP24* in AML using multidimensional analysis. To further explore the functional network and pathogenetic mechanism related to *FAAP24* in AML, we investigated its regulatory function in tumor immunity and its interaction with m6A RNA methylation and cuproptosis in AML. Our study fills the gap in discovering the biological function of *FAAP24* and uncovers a promising prognostic biomarker for AML.

## Methods and materials

The flow chart of this study is presented in Figure S1.

### Sources of data and processing methods

To explore the expression of *FAAP24* across tumors and normal controls, the gene expression levels of RNA sequencing (RNA-seq), including 33 types of cancer and 24 paracancerous tissues from TCGA (The Cancer Genome Atlas), were downloaded from the UCSC XENA website (https://xena.ucsc.edu/) [13]. A cohort combined with TCGA AML (n = 173), GTEx (Genotype-Tissue Expression, n = 337) and TARGET (Therapeutically Applicable Research To Generate Effective Treatments) AML (n = 196) was also collected to evaluate the differential expression of *FAAP24* between AML and normal whole blood controls. Subsequently, GSE65409, consisting of 8 normal controls and 30 AML samples, was used to verify the difference in *FAAP24* expression. The cohort of GDC TCGA AML (n = 151) was chosen to explore the prognostic value of *FAAP24* in AML. Moreover, we downloaded gene counts of the Beat AML cohort from the data viewer (www.vizome.org), and the summary of clinical data was acquired from a previous study [14, 15]. The toil method was performed to process the expression profile data, and all RNA-seq data were normalized as log2 (TPM + 1) before analysis. Then, a total of 446 Beat AML patients were investigated to determine the relationship of *FAAP24* expression with clinical features and validate the prognostic value of *FAAP24* in AML. Processed datasets of composite expression and compound activity (DTP NCI-60) were also downloaded to perform drug sensitivity analysis (https://discover.nci.nih.gov/cellminer/home.do) [16].

### Expression and survival analysis of ***FAAP24*** across cancers

To explore *FAAP24* expression across cancers, the Mann-Whitney U test was executed in each group. Box plots were used to exhibit the differential expression between tumors and normal tissues.

GEPIA2 (http://gepia2.cancer-pku.cn/) [17] was exploited to investigate the prognostic values of *FAAP24* in different types of tumors. Based on the median expression of *FAAP24* in different tumors, patients with each cancer were divided into two groups (*FAAP24*^high^ and *FAAP24*^low^). The Kaplan -Meier method with the log-rank test, accompanied by the Cox proportional hazards model, was applied to explore and visualize the prognostic values of *FAAP24* expression for overall survival (OS) across cancers.

### Development and validation of a nomogram containing ***FAAP24*** for AML prognosis

In the GDC TGCA AML cohort (n = 151), we performed univariate and multivariable survival analyses to investigate the prognostic role of the *FAAP24* gene. Subsequently, a nomogram was established for predicting AML prognosis using significant variables in the multivariable Cox survival model (P < 0.05). Receiver operator characteristic (ROC) and calibration curves at different time points were sketched to estimate the discrimination and calibration ability. Decision curve analysis (DCA) was also plotted to evaluate the clinical utility. Meanwhile, we used Beat AML to prove *FAAP24*’s impact on AML prognosis and the applicability of the nomogram. According to the median risk score of the nomogram, both TCGA and Beat AML were divided into two risk groups (high risk and low risk). Kaplan-Meier curves with hazard ratios (HRs) were then generated to demonstrate the nomogram’s efficiency. Finally, a Sankey diagram was created to display the ability of the nomogram to reclassify risk stratification from cytogenetics. All analyses were carried out by using the R packages “survival”, “survminer”, “rms”, “timeROC”, “dcurves”, “ggalluvial” and “ggplot2”.

### Analysis of ***FAAP24***-related expressed genes

According to the median expression of *FAAP24*, AML patients from TCGA were segregated into two groups (*FAAP24*^high^ and *FAAP24*^low^). The R package limma was adopted to search the differentially expressed genes (DEGs) between *FAAP24*^high^ and *FAAP24*^low^ patients. Pearson correlation analysis was used to screen the coexpressed genes of *FAAP24* in AML by using the LinkedOmics database (http://www.linkedomics.org/) [18]. Both DEGs and coexpressed genes were visualized using a volcano map, and then a Venn diagram was drawn to explore the intersecting genes between them for functional enrichment analysis using the R packages VennDiagram and ggplot2.

### Functional enrichment and immunological features in AML

Enrichment analyses of intersected genes, including GO (Gene Ontology) and KEGG (Kyoto Encyclopedia of Genes and Genomes) pathways, were carried out using the “clusteProfiler” R package. The hallmarks of tumors are of great significance for cancer research, and the enrichment in hallmark gene sets to a special phenotype may help to explain the mechanism related *to FAAP24* in AML. “H.all.v2022.1.Hs.symbols.gmt” was downloaded from the MSigDB website (http://software.broadinstitute.org/gsea/msigdb) as a reference gene set. Gene set enrichment analysis (ssGSEA) was executed based on a single sample gene in TCGA AML by means of the “GSVA” R package. Pearson coefficients were calculated to explore the potential phenotype of cancer linked to *FAAP24* expression.

The immune cycle in cancer reflects the anticancer immune response and consists of 7 steps. When these steps are activated to varying degrees, the destiny of cancer cells is determined at the same time [19]. We also performed ssGSEA to evaluate the anticancer immune cycle in TCGA AML based on individual gene expression. Then, Pearson correlation analyses were performed to scan the relationship between *FAAP24* and the enrichment of these steps of the anticancer immunity cycle.

Afterwards, we further calculated the immunological features of TCGA AML patients in the tumor microenvironment (TME) from individual gene expression using the R package “xCell” [20]. Spearman coefficients were determined first to identify *FAAP24*-related immune cells. Thereafter, patients were also split into two groups (*FAAP24*^high^ and *FAAP24*^low^) on account of the median expression, and the different abundances of immune cells were subsequently compared between these two groups through the Wilcoxon test. To explore whether *FAAP24* expression is modified by m6A and interacts with cuproptosis in AML, Spearman coefficients were also used to investigate the relationship of *FAAP24* with 23 m6A- and 10 cuproptosis-related genes [21, 22]. We visualized the results by using the R package “ggplot2”.

### Patient samples and Western blotting

We obtained whole blood samples from 5 healthy controls and bone marrow samples from 4 AML patients. The test of each sample was carried out in triplicate. Western blotting was performed as described previously [8]. *The FAAP24* primary antibody (GTX117277, United States) was purchased from GeneTex, and the GAPDH primary antibody (ab8245, United States) was obtained from Abcam.

### Cell Culture

AML line cells were purchased from the American Type Culture Collection (ATCC). The cells were cultured in 90% RPMI 1640 medium (Gibco, Thermo Fisher Scientific) with 10% fetal bovine serum (FBS), 100 U/mL penicillin and 100 mg/mL streptomycin at 37 °C in a humidified 5% CO2 incubator. The cells were harvested, washed, and resuspended in phosphate buffered saline (PBS).

### Real-time PCR

Four AML cell lines, HL-60, MV4-11, MOLM13 and U937, were harvested for expression analysis by quantitative PCR (qPCR) as previously described [23]. The following primers for *FAAP24* were used: *FAAP24*forwards, 5’- ATGGCTTGACACCAGACTTTT-3’, and reverse, 5’-TACTGGGCTCTTTGGTTTGC-3’. Relative levels of expression were computed using the 2^−ΔΔCt^ method with *FAAP24* expression in HL60 for normalization. The samples were run in triplicate parallel reactions.

### Drug sensitivity analysis and cell viability assay

Both expression levels and compound activity (DTP NCI-60, Z score) were downloaded in a processed format from the CellMiner website, and Pearson correlation analysis with a condition of |cor|>0.4 and P < 0.01 was performed to screen drug sensitivity associated with *FAAP24* expression.

Then, these four AML cell lines were used to confirm drug sensitivity in vitro. When cells were in the logarithmic growth period, AML cells were seeded in 96-well plates at a density of 6000 cells per well. After treatment with chelerythrine (MCE company, 0, 1, 10 µM) for 72 h, the cells were incubated with 10 µL of CCK-8 reagent (Dojindo) for 2 h at 37 °C. In each well, the optical density (OD) was measured using a microplate reader at 450 nm, and the OD values were recorded as the means ± SDs. Each assay was performed in triplicate. IC50 values were analysed in GraphPad Prism 5 software.

### Immunofluorescence staining

The distribution of *FAAP24* in HL-60, MV4-11, MOLM13 and U937 cells was determined as previously described [24]. Cells were fixed first with 2% paraformaldehyde at room temperature for 10 min. After blocking with 5% normal goat serum, cells were incubated with *FAAP24* antibody (Gene Tex) at 4 °C for 1 h and then incubated with secondary FITC-conjugated anti-rabbit Abs at 4 °C for 30 min. After washing with PBS, 100 ng/mL DAPI (Invitrogen) was added for nuclear staining at room temperature. Imaging was presented under a Zeiss LSM900 laser scanning confocal microscope (Zeiss, Germany).

### Statistical analysis

R software (v 4.2.1) was used to conduct statistical analysis. χ^2^ or fish tests were performed to analyse the differences in clinical features between the *FAAP24*^high^ and *FAAP24*^low^ groups, including age (> 60 vs. ≤60), sex, cytogenetic risk, classification of French-American-British (FAB), and gene mutations. The Mann-Whitney U test was performed to compare differences in continuous variables, such as white blood cell (WBC) count, percentage of blasts in peripheral blood (PB) or bone marrow (BM), hemoglobin (HB) and platelets (PLT). P values less than 0.05 were considered statistically significant.

## Results

### Expression and prognostic analysis of ***FAAP24*** across cancers

**Fig. 1:**
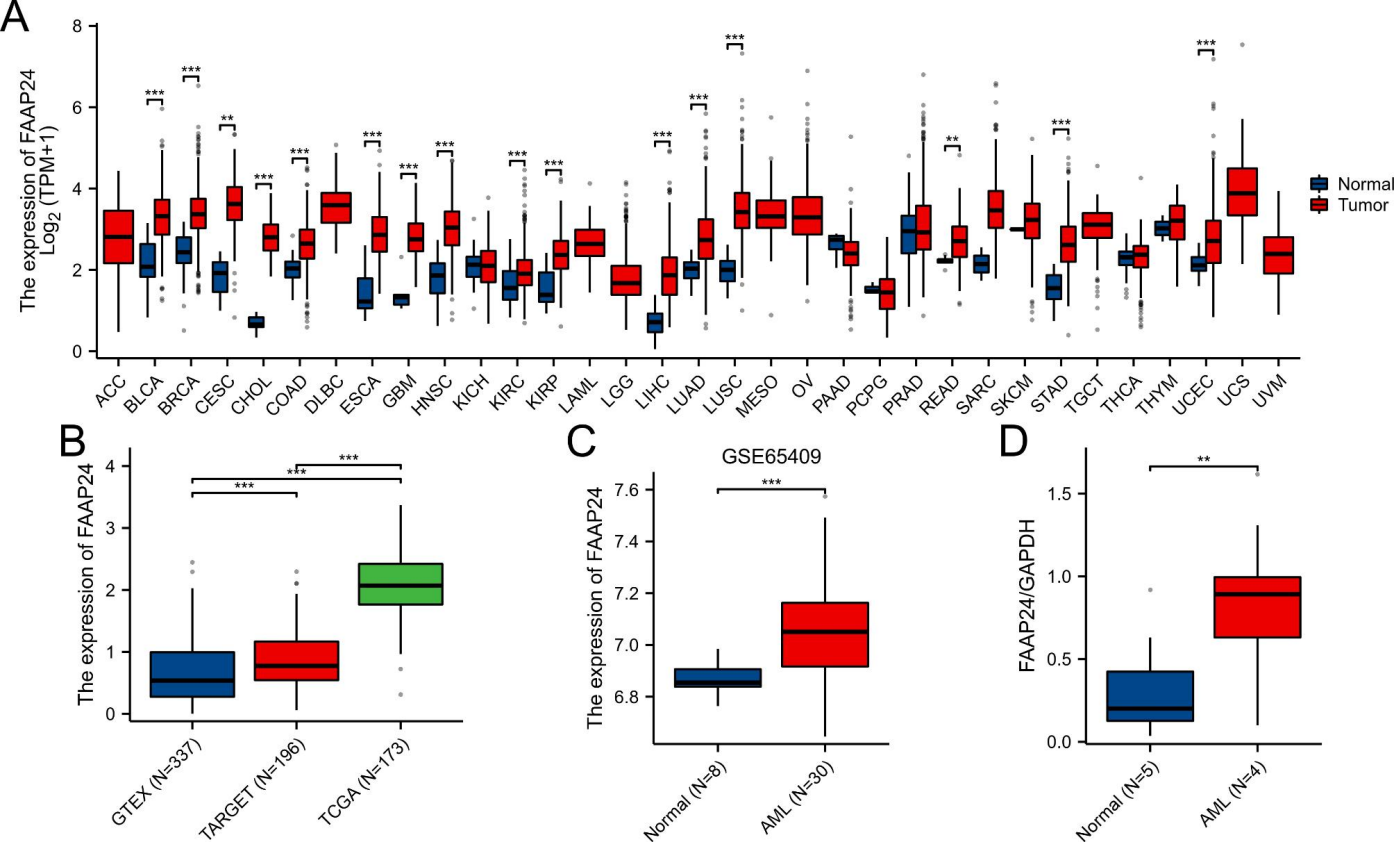
Differentially expressed analysis of *FAAP24* in tumor and normal tissues, analysed by using the Mann-Whitney U test. **(A)** The expression levels of *FAAP24* across 33 types of cancer and 24 paracancerous tissues using the TCGA database. (**B**) The levels of *FAAP24* expression across the GTEx, TCGA AML, and TARGET AML databases. (**C**) The expression of *FAAP24* in the GSE65409 cohort. (**D**) The ratio of FAAP24 to GAPDH between normal and AML samples, as determined by Western blotting. * indicates P < 0.05, ** indicates P < 0.01, *** indicates P < 0.001.

In pancancer analysis, the mRNA levels of *FAAP24* in 33 types of tumors were explored and are presented in Fig. 1A and Supplementary Table S1. The results showed that almost all cancers expressed higher *FAAP24* than normal tissues, including BLCA (bladder urothelial carcinoma), BRAC (breast cancer), and CESC (cervical cancer). Similarly, both TCGA AML (n = 173) and TARGET AML (n = 196) expressed higher *FAAP24* than normal controls (whole blood, n = 337) from the GTEx database (Fig. 1B). This differential expression was also confirmed in the GSE65409 cohort (Fig. 1C) and in our western blot results (Fig. 1D). These expressive features suggest that *FAAP24* may play a unique role in AML.

**Fig. 2:**
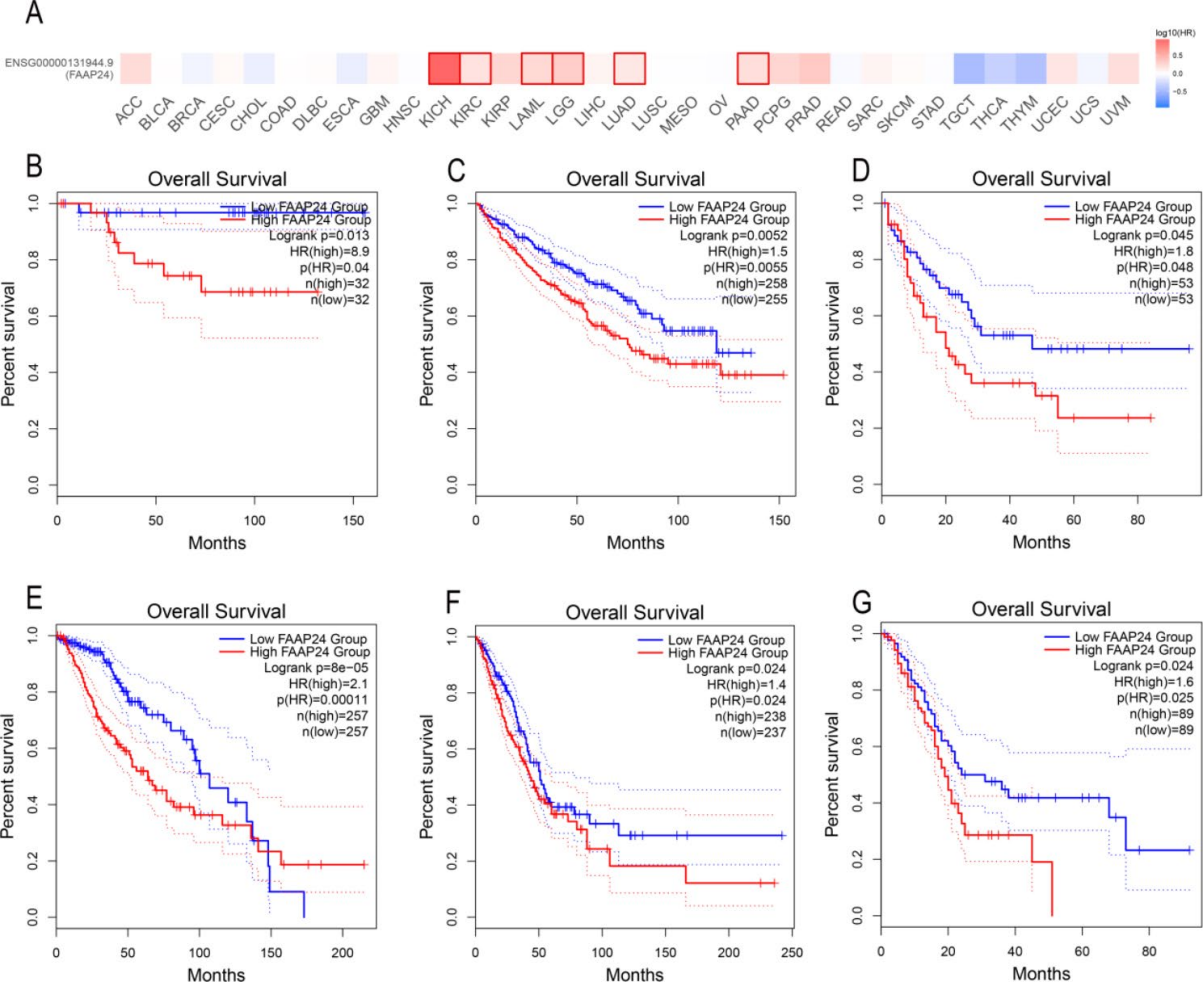
Survival analysis of *FAAP24* expression on overall survival (OS) in pancancer by GEPIA2 database. **(A)** Log10 transform of hazard ratio (HR) from Cox-regression model in different types of cancer. (**B**) KICH (kidney chromophobe), (**C**) KIRC (kidney renal clear cell carcinoma), (**D**) AML (acute myeloid leukemia), (**E**) LGG (brain lower grade glioma), (**F**) LUAD (lung adenocarcinoma), (**G**) PAAD (pancreatic adenocarcinoma). Dotted lines indicate the curves of the 95% confidence interval.

To analyse the effects of *FAAP24* on survival across cancers, patients with 33 types of tumors were split into two different risk groups based on the median value of *FAAP24* expression and analysed using the GEPIA2 database (Fig. 2A). The results showed that upregulation of *FAAP24* had poor prognostic value in KICH (kidney chromophore, Fig. 2B), KIRC (kidney renal clear cell carcinoma, Fig. 2C), AML (Fig. 2D), LGG (brain lower grade glioma, Fig. 2E), LUAD (lung adenocarcinoma, Fig. 2F), and PAAD (pancreatic adenocarcinoma Fig. 2G) (P < 0.05).

### Independent prognostic value of ***FAAP24*** in AML

Survival curves with log-rank tests and Cox proportional analyses inferred that high *FAAP24* expression presented a significantly inferior OS than low *FAAP24* expression in AML from the GEPIA2 database. Subsequently, GDC TCGA AML (n = 151) was further used to explore the relationship of *FAAP24* with clinical features and confirm the prognostic role of *FAAP24* in AML.

**Fig. 3:**
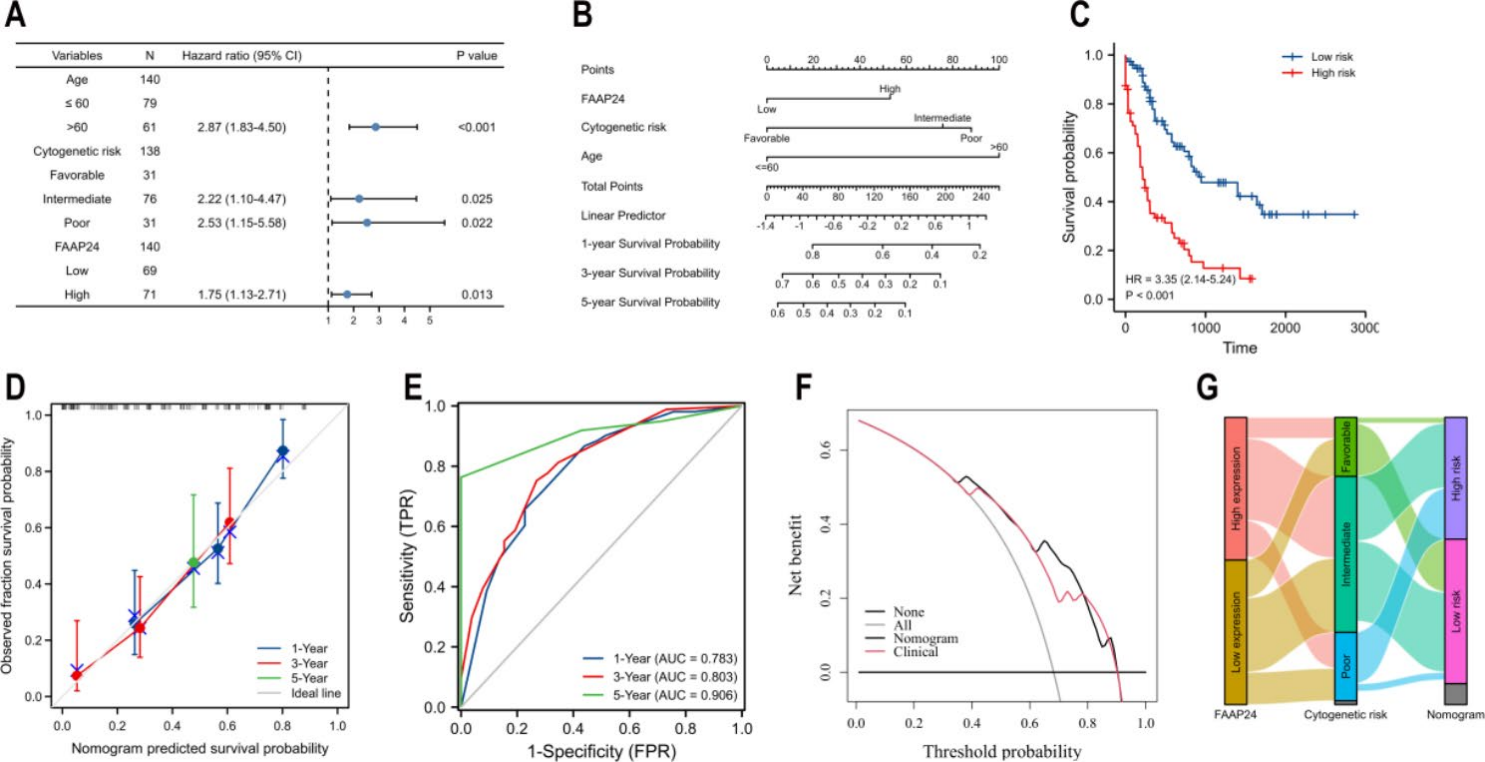
Nomogram construction containing *FAAP24* expression in TCGA AML. (**A**) Forest plot showing multivariate Cox analysis for AML OS in TCGA. (**B**) Development of a nomogram including three independent prognostic variables (a line is plotted from each variable axis upwards to calculate the value of points for each variable. An individual patient’s prognostic score was determined by the sum of these values, which is located on the total points axis, and a vertical line is sketched downwards to the different survival axes to estimate the probability of survival). (**C**) Survival analysis of the high- and low-risk groups stratified by the nomogram, analysed by the Cox regression model and visualized by using the Kaplan-Meier method. (**D**) Calibration curve of the nomogram at 1, 3, and 5 years. (**E**) ROC curves of the nomogram at 1, 3, and 5 years. (**F**) DCA plot at 5 years for the nomogram and clinical model (age plus cytogenetic risk). (**G**) Sankey curve of transitions from three categories of cytogenetic risk to reclassification of the nomogram.

The clinical characteristics between the *FAAP24*^high^ and *FAAP24*^low^ groups are summarized in Table 1. In the results, more M5 and male patients were assigned to the *FAAP24*^high^ group. Univariate Cox proportional analysis showed that *FAAP24* expression (high vs. low, P = 0.002), age (> 60 vs. ≤60, P < 0.001) and cytogenetic risk (intermediate vs. favorable, P = 0.002; poor vs. favorable, P < 0.001) significantly affected the OS of AML (Supplementary Table S2). An independent prognostic role for *FAAP24* expression in AML was confirmed by a multivariate model (HR = 1.75, P = 0.013, Fig. 3A and Supplementary Table S2).

**Table 1:** Clinical characteristics of patients in TCGA AML cohort

Characteristic	Low expression of *FAAP24*	High expression of *FAAP24*	P value
n	75	76	
Gender, n (%)			**0.028**
Female	41 (27.2%)	27 (17.9%)	
Male	34 (22.5%)	49 (32.5%)	
Race, n (%)			0.670
Asian	1 (0.7%)	0 (0%)	
Black or African American	7 (4.7%)	6 (4%)	
White	66 (44.3%)	69 (46.3%)	
Age, n (%)			0.357
≤ 60	47 (31.1%)	41 (27.2%)	
> 60	28 (18.5%)	35 (23.2%)	
Cytogenetic risk, n (%)			0.206
Favorable	20 (13.4%)	11 (7.4%)	
Intermediate	38 (25.5%)	44 (29.5%)	
Poor	17 (11.4%)	19 (12.8%)	
FAB classifications, n (%)			**0.002**
M0	10 (6.7%)	5 (3.3%)	
M1	19 (12.7%)	16 (10.7%)	
M2	20 (13.3%)	18 (12%)	
M3	13 (8.7%)	2 (1.3%)	
M4	11 (7.3%)	18 (12%)	
M5	2 (1.3%)	13 (8.7%)	
M6	0 (0%)	2 (1.3%)	
M7	0 (0%)	1 (0.7%)	
WBC (x10^9/L), median (IQR)	14 (3, 42.25)	28.5 (7, 54)	0.062
BM blasts (%), median (IQR)	37 (6, 67.5)	40.5 (10.75, 63.25)	0.537
PB blasts (%), median (IQR)	69 (48, 86)	72 (51, 84.25)	0.542

WBC: white blood cells, BM: Bone marrow, PB: Peripheral Blood

### Development of a predicted nomogram containing ***FAAP24*** in TCGA AML

A nomogram (Fig. 3B) was developed for predicting AML OS using a multivariate Cox regression model. Evidence of age older than 60 was assigned 100 points, while the score was 0 points if age ≤ 60. The favorable risk of cytogenetics was regarded as the reference and assigned a 0 point, while 76 and 88 points were given for intermediate and poor risk of cytogenetics, respectively. Similarly, zero points was assigned to the low expression of *FAAP24*, while fifty-three points was given for the evidence of high *FAAP24* expression. The prognostic score for each patient was determined by the sum of the weight integral of each variable. According to the median prognostic risk score of the nomogram, patients with high prognostic scores had inferior OS than patients with low risk scores (HR = 3.35, P = 0.002, Fig. 3C). Calibration plots showed good performances at 1, 3 and 5 years (Fig. 3D), and the ROC curves also demonstrated good discrimination (Fig. 3E). At 1, 3 and 5 years, these values are 0.783, 0.803 and 0.906, respectively.

When compared with the clinical model combined with age and cytogenetic risk only, DCA showed that the nomogram presented various benefits at 5 years (Fig. 3F). Moreover, the prognosis of AML predicted by the nomogram exhibited more benefits than either the intervention-all or intervention-none strategy at the different threshold points. In addition, the cytogenetic risk of AML could be further reclassified on the basis of the nomogram (Fig. 3G).

### Validation of the nomogram containing ***FAAP24*** in beat AML

**Fig. 4:**
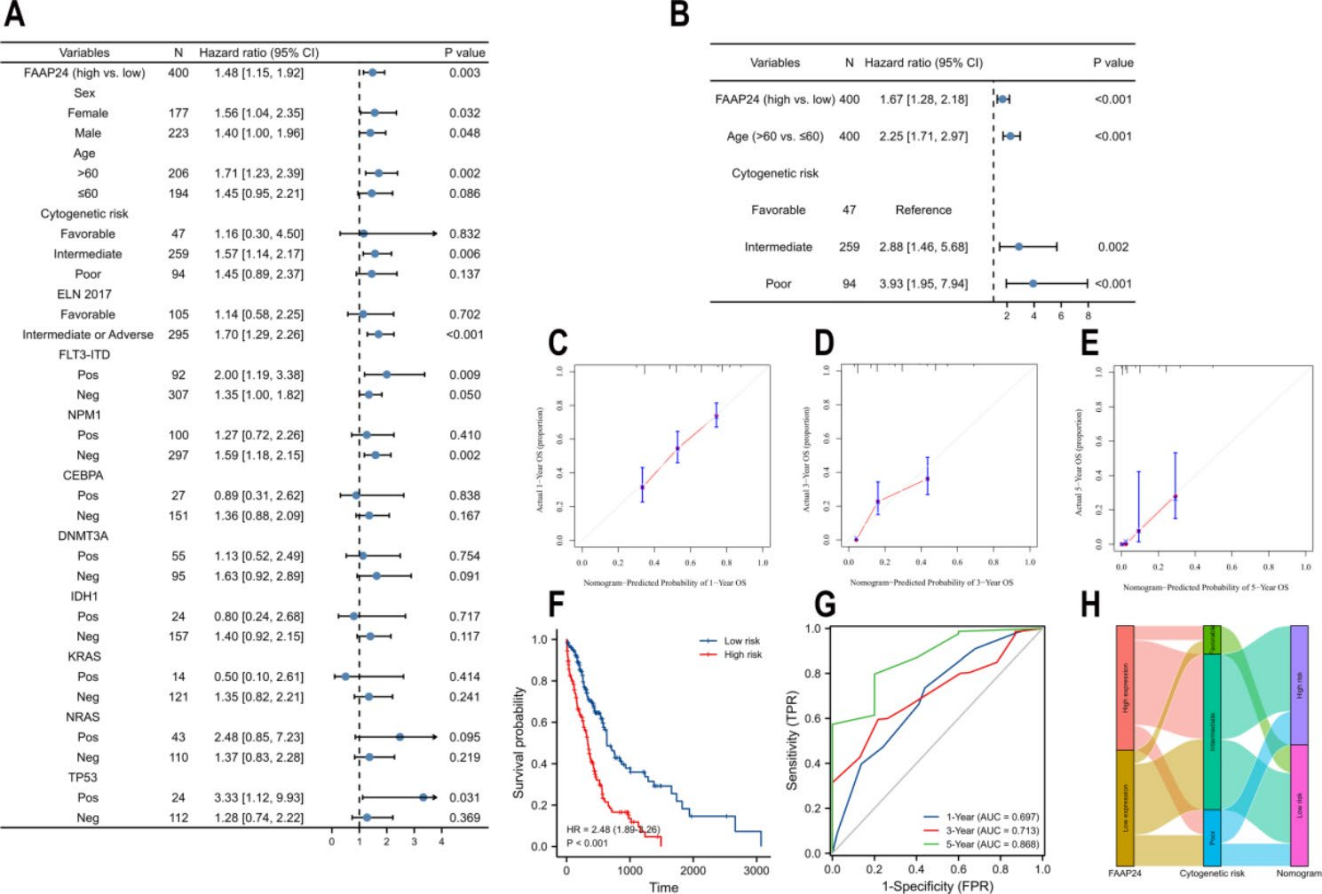
External validation of the nomogram containing *FAAP24* (A) Subgroup analysis of *FAAP24* expression for AML OS in the Beat cohort. (**B**) Forest plot for the multivariate survival model in the Beat AML cohort. Calibration curves of external validation for the nomogram at 1 (**C**), 3 (**D**), and 5 years (**E**). (**F**) Survival analysis between two different risk scores stratified by the nomogram in the validation dataset. (**G**) ROC curves of external validation. (**H**) Sankey curve of transitions from cytogenetic risk to two risk categories of the nomogram in external validation

To confirm the poor prognostic value of *FAAP24* in AML, an independent AML cohort (n = 446) was downloaded and analysed, and its clinical features are presented in Supplementary Table S3. Univariate Cox proportional analysis showed that *FAAP24* expression (high vs. low, P = 0.003), age (> 60 vs. ≤60, P < 0.001), cytogenetic risk (intermediate vs. favorable/poor vs. favorable, P < 0.001), and risk recommendation of ELN (European LeukemiaNet) 2017 (Intermediate or Adverse vs. Favorable, P < 0.001) were significantly related to OS in AML (Supplementary Table S4). Subgroup survival analysis (Fig. 4A) implied that *FAAP24* may play an important role in patients with age > 60 (P = 0.002), cytogenetic risk of intermediate (P = 0.006), Intermediate or Adverse of ELN 2017 risk recommendation (P < 0.001), patients with *NPM1*^neg^ mutation (P = 0.002), and patients with *TP53* mutation (P = 0.031). An independent prognostic value of *FAAP24* was also confirmed in Beat AML (HR = 1.67, P < 0.001, Fig. 3B and Supplementary Table S5).

In Beat AML, calibration plots of the external validation verified the practicability of the predicted nomogram containing *FAAP24* at different times (Fig. 4C, D and E). An inferior OS was presented in high-risk patients (HR = 2.48, P < 0.001, Fig. 4F). The ROC curves of external validation also confirmed good discrimination (Fig. 4G), with values of 0.697, 0.713 and 0.868 at 1, 3 and 5 years, respectively. Similar to TCGA AML results, reclassification of cytogenetic risk could be performed based on the predicted nomogram (Fig. 3H).

### Analysis of ***FAAP24***-related genes in AML

**Fig. 5:**
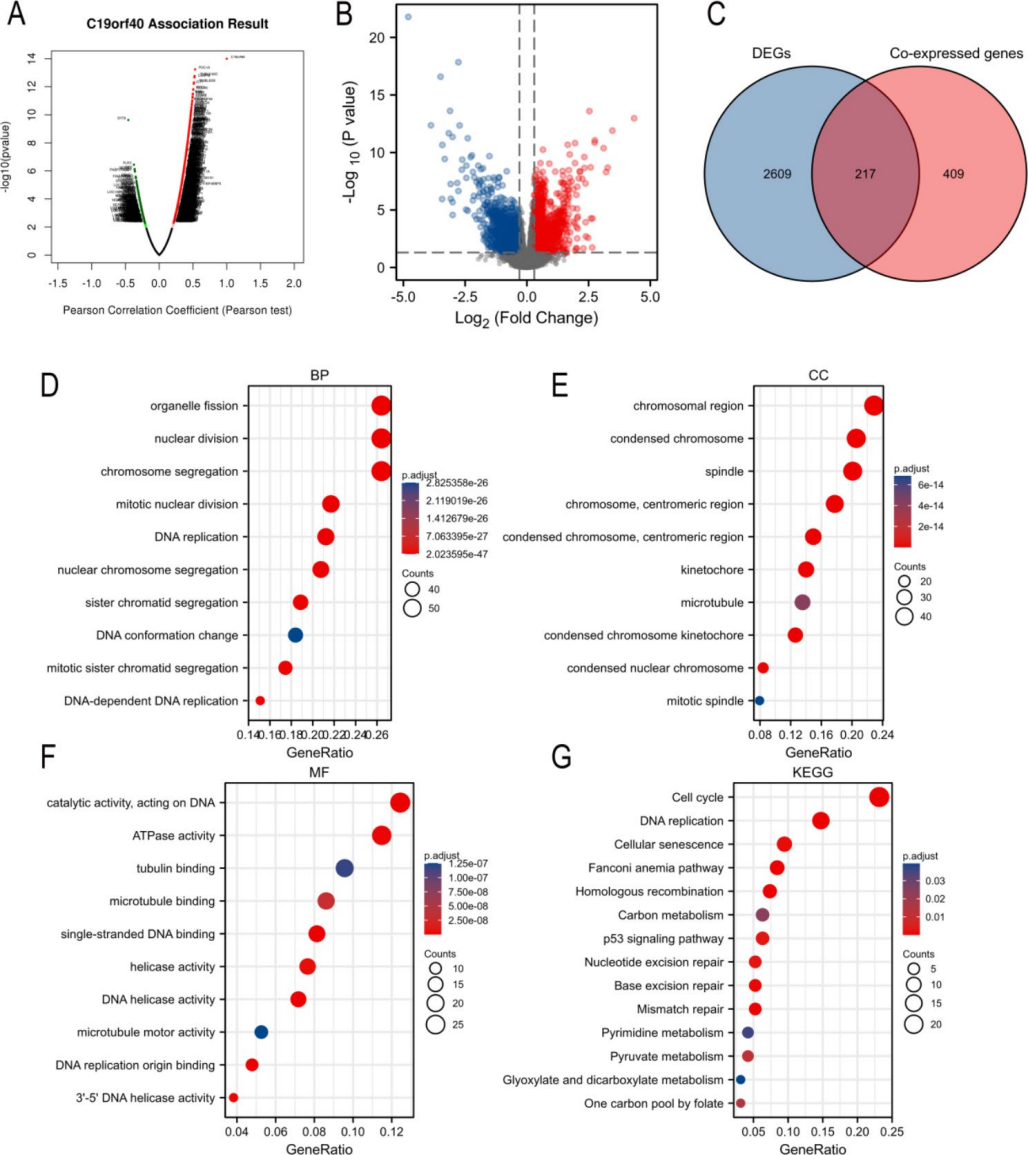
*FAAP24-*related genes and enrichment analysis. **(A)** Coexpressed genes associated with *FAAP24* in AML, analysed by using LinkedOmics and visualized by Volcano plot. (**B**) Volcano curve for DEGs between the *FAAP24*^high^ and *FAAP24*^low^ patients. (**C**) Venn diagram showing the intersecting genes. Bubble charts of biological process (BP) (**D**), cellular component (CC) (**E**), and molecular functions (MF) (**F**) in GO analysis. KEGG analysis of intersecting genes (**G**)

To better explore the underlying pathogenic mechanism of *FAAP24* in AML, the coexpressed genes of *FAAP24* were screened out first in TCGA-AML patients using the LinkedOmics database (Fig. 5A). A total of 586 genes were positively coexpressed with *FAAP24* in AML, while 40 genes were negatively coexpressed with *FAAP24* (Supplementary Table S6, |cor|>0.3, FDR < 0.05). Then, we also identified 2826 DEGs between *FAAP24*^high^ and *FAAP24*^low^ in AML (Fig. 5B, Supplementary Table S7), including 1229 upregulated (log2 FC > 0.3, P < 0.05) and 1597 downregulated protein-coding genes (log2 FC<-0.3, P < 0.05). Subsequently, a total of 217 *FAAP24*-related genes overlapped between coexpressed genes and DEGs of *FAAP24* (Fig. 5C), consisting of 207 upregulated genes and 10 downregulated genes, which were included for further functional enrichment analysis.

### Biological function of ***FAAP24*** in AML

To investigate the potential biological function of *FAAP24* in AML, we performed GO/KEGG analyses on the 217 overlapping genes (Supplementary Table S8) using bubble charts to present the top enriched items. The main sets of BP (biological process) were organelle fission, nuclear division and chromosome segregation (Fig. 5D), and chromosomal region was the major list of CC (cellular component) (Fig. 5E). The molecular functions (MF) of *FAAP24* and its partner genes were primarily involved in catalytic activity that acts to modify DNA (Fig. 5F).

KEGG analyses (Fig. 5G) suggested that *FAAP24* and its related genes were mainly involved in DNA damage and repair pathways. In addition to the cell cycle, DNA replication and cellular senescence, these genes were also involved in regulating the metabolic processes of AML, such as pyruvate, carbon, pyrimidine, glyoxylate and dicarboxylate metabolism. These results implied the potential biological role of *FAAP24* in leukemogenesis.

**Fig. 6:**
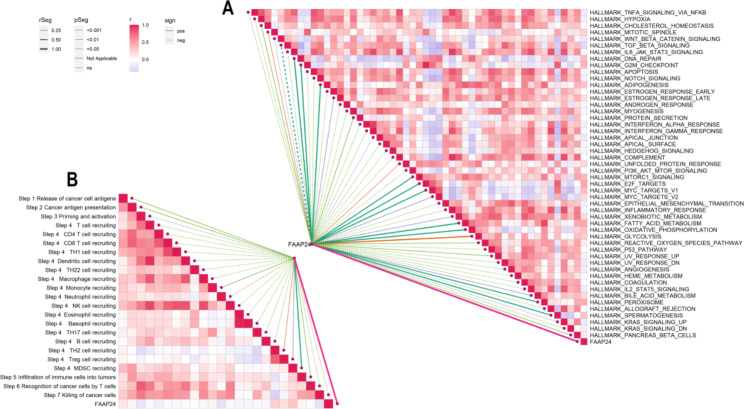
Roles of *FAAP24* expression in cancer phenotypes and the anticancer immune cycle (**A**) Relationship of *FAAP24* expression with the enrichment scores (ESs) of the hallmark gene set. (**B**) Correlations between *FAAP24* expression and different steps of the anticancer immunity cycle

Moreover, we also explored the correlation between *FAAP24* and the enrichment scores of the tumor pathway from the hallmark gene set (Fig. 6A, Supplementary Table S9). *FAAP24* was positively associated with proliferation and the cell cycle, including E2F TARGETS (r = 0.452), FATTY ACID METABOLISM (r = 0.436), PEROXISOME (r = 0.424), G2M CHECKPOINT (r = 0.376), DNA REPAIR (r = 0.363), MTORC1 SIGNALING (r = 0.358) and MYC TARGETS V1 (r = 0.346), but negatively associated with TGF BETA SIGNALING (r=-0.285).

### ***FAAP24*** shapes an immunosuppressive TME in AML

To investigate the role of *FAAP24* in the immune regulation of AML, the relationship between *FAAP24* and the antitumour immune response is presented in Fig. 6B and Supplementary Table S10. Different steps of the anticancer immune cycle showed that higher *FAAP24* expression in AML strongly correlated with a stronger recruitment of MDSCs (r = 0.314), TH2 cells (r = 0.239) and Treg cells (r = 0.175) but was negatively related to the processes of killing cancer cells (r=-0.226) and priming of antitumour immune response (r=-0.200). All these discoveries supported why high *FAAP24* expression is often related to poor prognosis in AML.

**Fig. 7:**
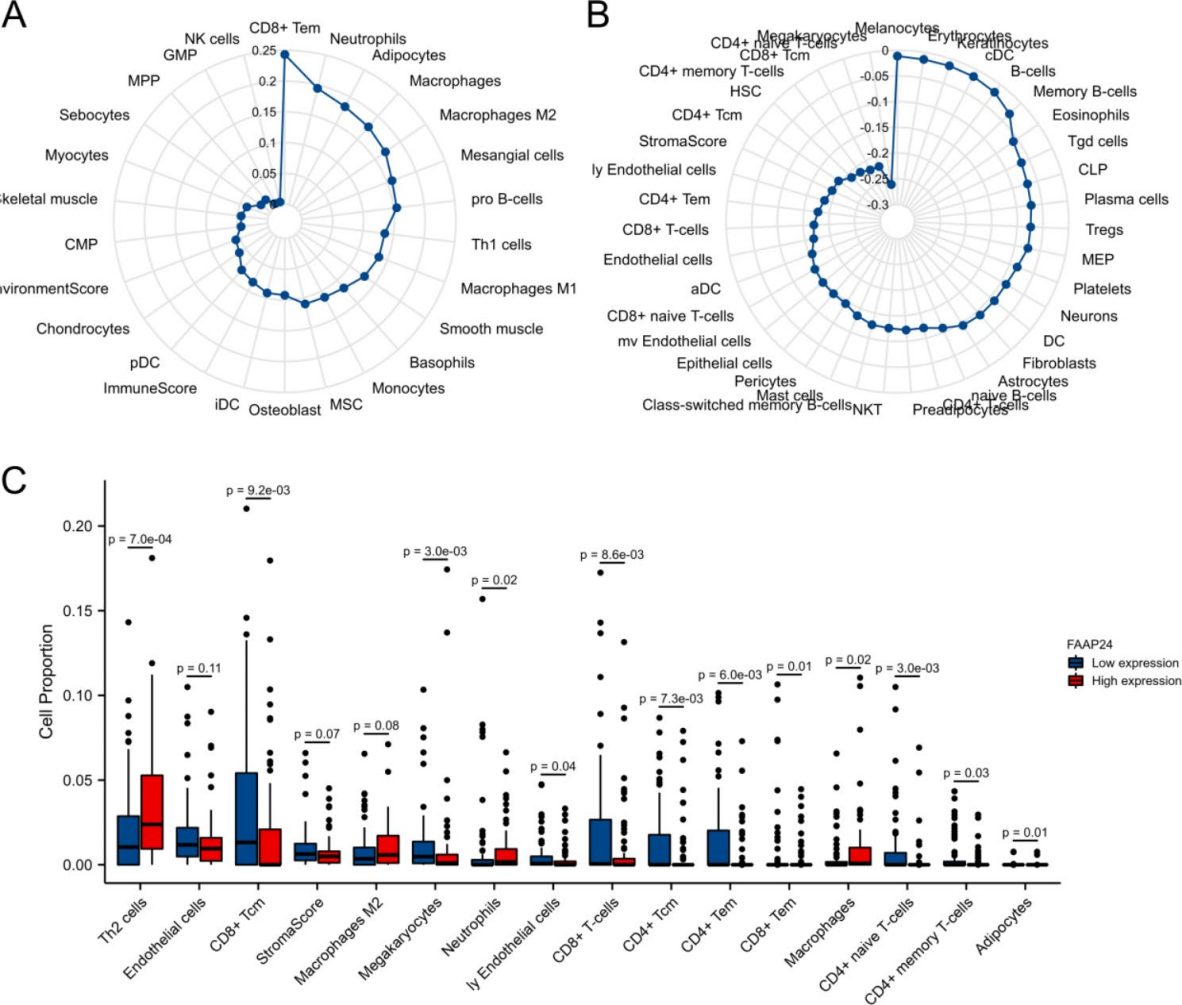
Immunological features associated with *FAAP24* expression in the tumor microenvironment **(A)** Immune cells with different degrees of positive correlation with *FAAP24* expression were analysed by using the “xCell” R package and visualized by radar plot. (B) Immune cells with different degrees of negative correlation with *FAAP24* expression. (C) The difference in the top-related immune components (n = 16) between *FAAP24*^high^ and *FAAP24*^low^ expression patients, analysed by the Mann-Whitney U test

Because of the adverse antitumour immune response, xCell was also used to further evaluate the TME induced by *FAAP24* in AML. The results demonstrated that *FAAP24* was positively (Fig. 7A) linked to Th2 cells (r = 0.299) and M2 macrophages (r = 0.171) but negatively (Fig. 7B) related to CD4 + naive T cells (r=-0.220), CD8 + Tcm cells (r=-0.220), CD4 + memory T cells (r=-0.213), CD4 + Tcm cells (r=-0.194), CD4 + Tem cells (r=-0.178) and CD8 + T cells (r=-0.171). When compared with the *FAAP24*^low^ group, the *FAAP24*^high^ group displayed low-level infiltration of immune cells, including many subtypes of CD8 + and CD4 + cells (Fig. 7C), but more Th2 cells and macrophages (P < 0.05). Although not statistically significant, the *FAAP24*^high^ group still presented a higher tendency of M2 macrophage cells than the *FAAP24*^low^ group (P = 0.08). These findings suggested that *FAAP24* shapes an immunosuppressive TME in AML, which may help to promote leukemia progression.

### ***FAAP24*** interacted with m6A RNA methylation and cuproptosis in AML

**Fig. 8:**
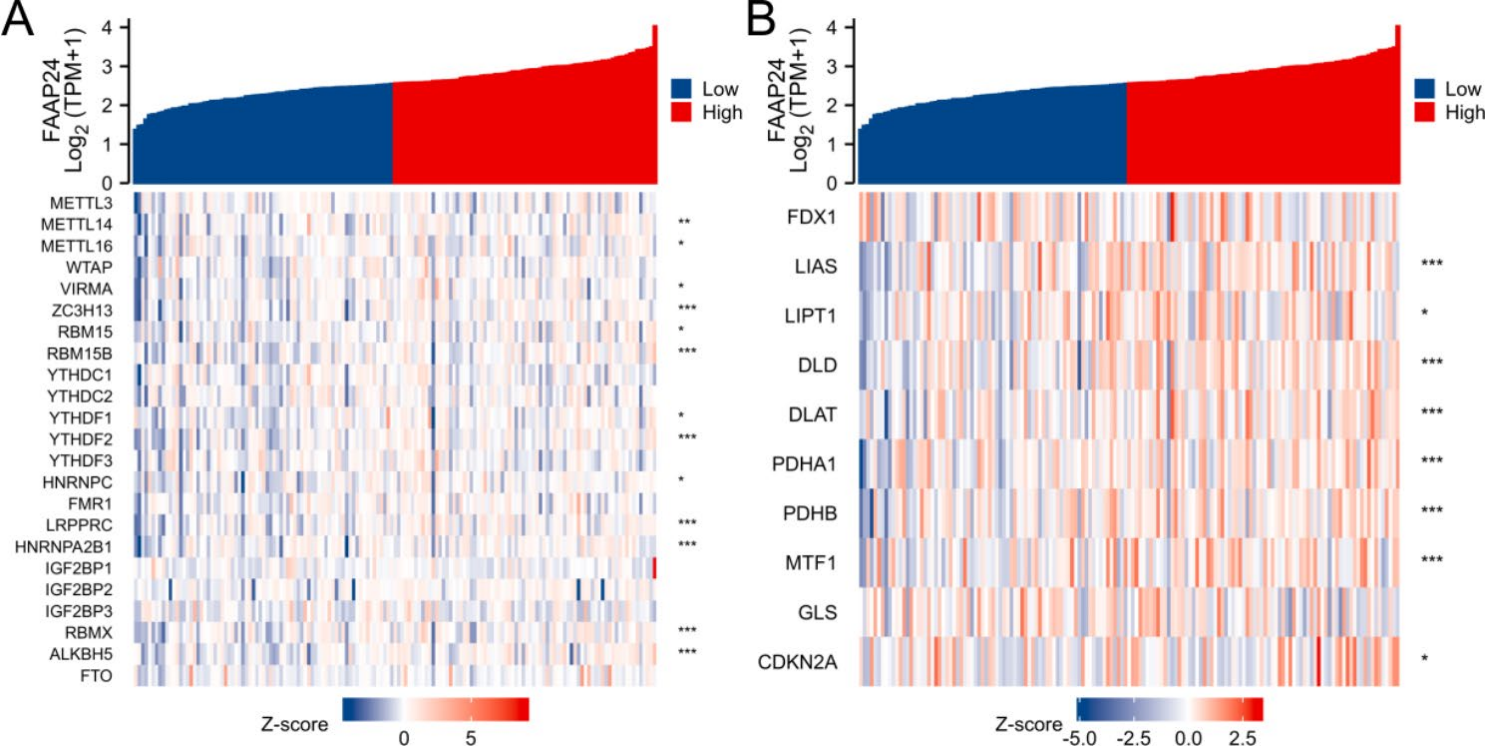
Heatmap of correlation analysis between *FAAP24* expression and m6A- (**A**) and cuproptosis-related (**B**) genes in AML, analysed by using Spearman coefficients

To determine whether *FAAP24* expression is mediated by m6A modification, the relationship of *FAAP24* with m6A-related genes was probed in AML. In Fig. 8A, the expression level of *FAAP24* was positively related to 13 m6A-related genes, including METTL14 (r = 0.254), METTL16 (r = 0.192), VIRMA (r = 0.207), ZC3H13 (r = 0.290), RBM15 (r = 0.202), RBM15B (r = 0.291), YTHDF1 (r = 0.163), YTHDF2 (r = 0.316), HNRNPC (r = 0.207), LRPPRC (r = 0.328), HNRNPA2B1 (r = 0.291), RBMX (r = 0.274), and ALKBH5 (r = 0.360). Moreover, 8 of 10 cuproptosis-related genes were significantly associated with *FAAP24* (Fig. 8B): LIAS (r = 0.343), LIPT1 (r = 0.193), DLD (r = 0.368), DLAT (r = 0.307), PDHA1 (r = 0.387), PDHB (r = 0.368), MTF1 (r = 0.291), and CDKN2A (r = 0.185). These conclusions implied that *FAAP24* is associated with m6A modification and cuproptosis in AML.

### High ***FAAP24*** expression is associated with chelerythrine resistance

**Fig. 9:**
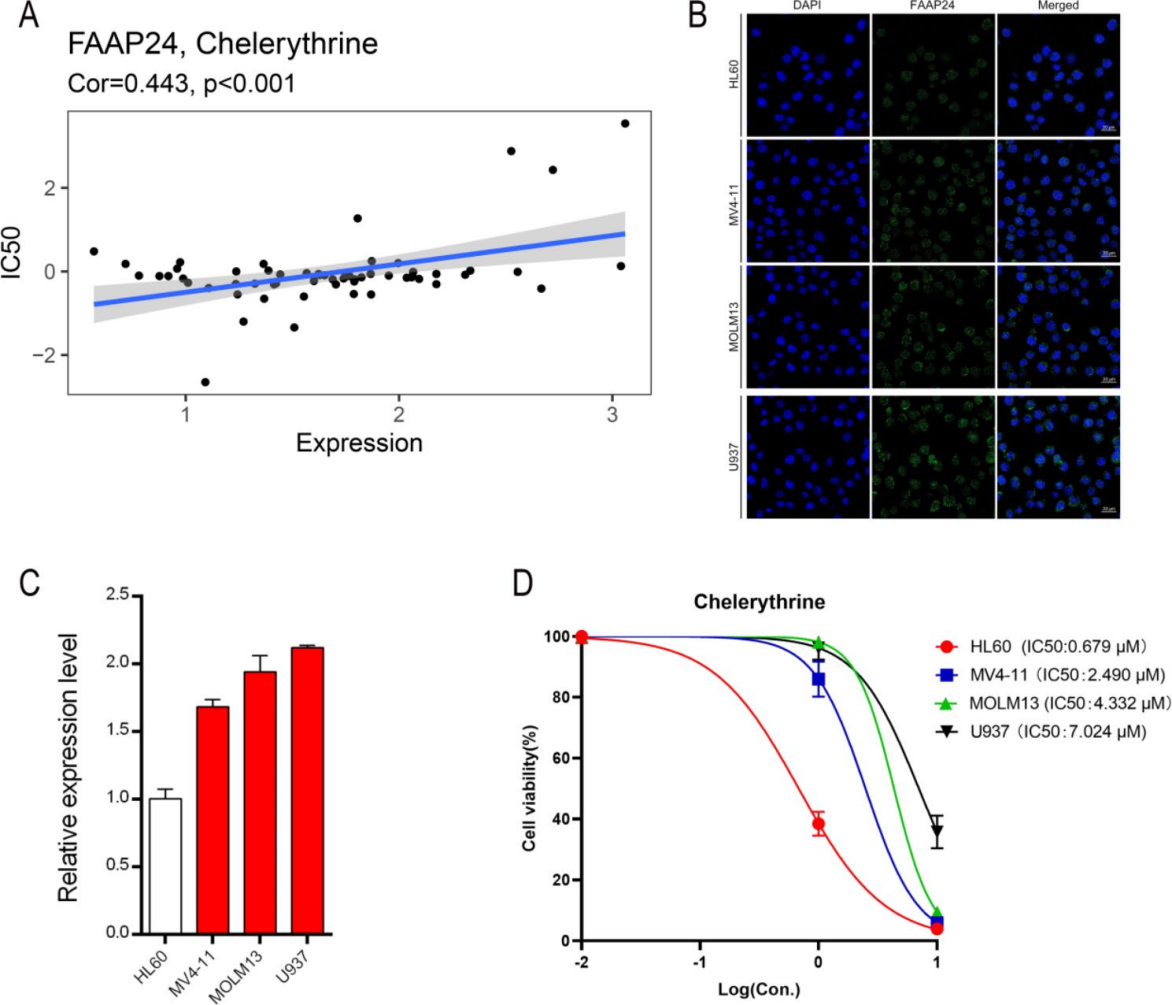
Drug sensitivity analysis of *FAAP24* expression and verification in vitro. (A) Correlation analysis of *FAAP24* expression with chelerythrine sensitivity using datasets from CellMiner. (**B**) Fluorescent location labelling of *FAAP24* in four AML cell lines. (**C**) Quantitative polymerase chain reaction (qPCR) of *FAAP24* expression in four AML cell lines. (**D**) Half maximal inhibitory concentration (IC50) of chelerythrine in four AML cell lines

Using the CellMiner database, drug sensitivity analysis showed that chelerythrine was meaningfully correlated with *FAAP24* expression (Fig. 9A, r = 0.443, P < 0.001). Then, 4 AML cell lines were used to confirm the correlation of *FAAP24* with chelerythrine sensitivity in vitro. In these 4 cell lines, *FAAP24* mainly localized to the nucleus and was visualized using fluorescent labelling (Fig. 9B). The qPCR results presented a gradual increasing trend in *FAAP24* expression in HL-60, MV4-11, MOLM13 and U937 cells (Fig. 9C). Drug sensitivity showed that high *FAAP24* expression was associated with chelerythrine resistance, with IC50 values of 0.679 µM, 2.490 µM, 4.332 µM, and 7.024 µM, respectively (Fig. 9D).

## Discussion

AML is a highly heterogeneous stem/progenitor cell disease that results from chromosome translocation and mutation of hematopoietic proliferation- and differentiation-related genes. Recent studies have indicated that some genetic abnormalities and specific chromosomal translocations in AML are associated with deficiencies in DNA repair pathways [25]. Among the canonical DNA damage repair pathways, the Fanconi anemia pathway is a new player that intersects with many other repair processes to respond to interstrand crosslink DNA lesions and thus to maintain chromosome stability [26]. To date, at least 22 FA complementation groups and many other interacting proteins have been identified in the FA pathway. Although it has been discovered that genetic mutations or abnormal expression in components of the FA pathway play important roles in the development of AML, treatment strategies targeting this pathway have made little progress.

As a component of the FA core complex, *FAAP24* functions in recognizing and binding to damaged DNA and stalled replication folks. According to the GTEx and TCGA databases, high expression of *FAAP24* in AML may promote the development of AML and thus has the potential to be a novel prognostic biomarker. It is yet to be determined whether *FAAP24* acts as a tumor suppressor gene and how it works.

We further analysed the association between *FAAP24* and OS across cancers using GEPIA2, and *FAAP24* expression was significantly associated with survival in patients with six cancers. To verify the prognostic value of *FAAP24*, we analysed its relationship with prognosis in the TCGA AML and Beat AML cohorts. The results revealed significant prognostic values of *FAAP24* expression in subgroups, including age, cytogenetic risk, *NPM1*^neg^ mutation and *TP53* mutation. All of these results confirmed the independent prognostic value of *FAAP24* expression for OS in AML. It is worth noting that, as we mentioned before, *FAAP24* is expressed at high levels in AML, and the prognosis is poor in the *FAAP24*^high^ group. This phenomenon might be caused by the DNA damage repair function of this gene. DNA damage caused by anticancer drugs is quickly recognized and repaired by the overexpression of *FAAP24*, leading to drug resistance.

The enrichment results confirmed that *FAAP24* was closely linked to the DNA damage repair process and cell cycle-related process in AML. The mechanisms of DNA repair proteins are often unclear and can be altered during the development of tumors. *FANCM-FAAP24* is important for *ATR*-mediated checkpoint signaling in response to replication stress [27]. These results suggest that abnormal expression of *FAAP24* may impact the normal proliferation, differentiation, cell apoptosis and hematopoietic function of bone marrow. The role of genetic and epigenetic changes in regulating cancer development and immune tolerance must be further explored, as well as the protein-protein interactions of *FAAP24*.

Many immunosuppressive factors are present in the TME, which is also an important factor hindering the benefits of cancer treatment. This immunosuppressive effect is attributed to many substances produced by tumors and immune cells. Additionally, the TME is packed with Tregs, MDSCs, TAMs and TANs and promotes tumor survival through the secretion of TGF-β, IL-10, nitric acid, and IDO (indoleamine 2,3 dioxygenase) [28]. The relationship between *FAAP24* and antitumour immune cells in AML was explored. The results showed that higher *FAAP24* expression in AML strongly correlated with a stronger recruitment of MDSCs, TH2 cells and Treg cells but was negatively related to the process of killing cancer cells and priming of immune response. The proliferation and differentiation of MDSCs into TAMs is induced by AML blasts, thus further inhibiting their immunogenicity [29]. On the other hand, increased Treg cells mediate T-cell suppression. Meanwhile, the high expression of *FAAP24* is positively related to M2 macrophages, which are immunosuppressive and promote tumor progression by facilitating angiogenesis and tissue phagocytosis [30]. All these factors lead to the promotion of leukemia progression, which explains the poor prognosis of high *FAAP24* expression in AML in our study. Further exploration of the strategies of tumor immune escape and the regulatory factors of M2 macrophages that are related to *FAAP24* in AML would provide immunologic insights into novel treatments.

Considering the close correlation between *FAAP24* and m6A methylation and cuproptosis-related genes in our results, further experiments could be performed to verify their interactions and their mechanism of promoting the development of AML. For example, *RBM15*, which modifies m6A targets in the Notch signaling pathway, is highly expressed in AML [31]. Other m6A genes that have been found to play oncogenic roles in AML include *WTAP*, *METTL3*, *METTL14*, *FTO*, *ALKBH5*, *IGF2BP1*, and *PRMT6* [32–34]. Among them, *METTL14*, *RBM15* and *ALKBH5* were significantly associated with *FAAP24*, which enhanced our understanding of the mechanism of *FAAP24* in AML. The role of cuproptosis in AML is rarely studied. It was reported that 3 cuproptosis-related genes, *GCSH*, *LIPT1*, and *DLAT*, have independent and significant prognostic value in AML [35]. In our research, *LIPT1* and *DLAT* were significantly associated with *FAAP24*. Our research provides evidence that m6A methylation and cuproptosis are related to AML and that *FAAP24* might play an important role in these processes. Further investigation should be performed to explore the possible molecular pathways.

By retrieving data from the NCI-60 cell line and 4 AML cell lines, our study illustrated that increased *FAAP24* expression levels were associated with chelerythrine resistance. Chelerythrine is reported to inhibit proliferation and induce apoptosis through several signaling pathways [36]. Although it is currently the focal point in antitumour research and its effect has been approved in prostate cancer, cervical cancer, gastric cancer cells, non-small cell lung carcinoma, renal cell carcinoma, etc. [37], little research has been performed on AML. The findings of our study provide new strategies for treating AML, and further studies are needed to confirm the pharmacological mechanism of chelerythrine in this disease.

Our study aimed to investigate the prognostic value of *FAAP24* in AML, its relationship with tumor-infiltrating immune cells, and its associated pathways. It was also confirmed that *FAAP24* expression was associated with poor prognosis and suppressed TME in AML. Finally, drug sensitivity analyses suggested a correlation between *FAAP24* expression and chelerythrine resistance, which could be a novel treatment strategy. The results validated the value of *FAAP24* as a prognostic biomarker in AML and identified significant areas for further research.

## Conclusions

In summary, we identified significant areas for further exploration and confirmation of the value of *FAAP24* as a prognostic biomarker in AML.

## Electronic supplementary material

Below is the link to the electronic supplementary material.

Supplementary Material 1

Supplementary Material 2

## Data Availability

Related datasets of TCGA, TARGET, GTEx, GSE65409, Beat AML and DTP NCI-60 could be downloaded from the UCSC XENA website, cBioPortal website and NIH websites. The data that support the findings of this study are available from the corresponding author upon reasonable request.

## Abbreviations

AML: Acute myeloid leukemia
FAAP24: FA-associated protein 24
TP53: Tumor protein p53
FANCM: Fanconi anemia complementation group M
FA: Fanconi Anemia
ssDNA: single-stranded DNA
TCGA: The Cancer Genome Atlas
GTEx: Genotype-Tissue Expression
GEPIA: Gene Expression Profiling Interactive Analysis
GDC: Genomic Data Commons
ROC: Receiver operator characteristic
DCA: Decision curve analysis
HRs: Hazard ratios
DEGs: expressed genes
GO: Gene Ontology
KEGG: Kyoto Encyclopedia of Genes and Genomes
TME: Tumor microenvironment
ssGSEA: Single sample gene set enrichment analysis
FITC: Fluorescein Isothiocyanate
DAPI: 4’,6-Diamidino-2-phenylindole
ACC: adrenocortical cancer
BLCA: bladder urothelial carcinoma
BRAC: breast cancer
CESC: cervical cancer
KICH: Kidney Chromophobe
KIRC: Kidney renal clear cell carcinoma
LGG: Brain Lower Grade Glioma
LUAD: Lung adenocarcinoma
PAAD: Pancreatic adenocarcinoma
OS: Overall survival
ELN: European Leukemia Net
BP: biological process
CC: cellular component
MF: molecular functions
Tcm: Central memory T-cell
Th2: T helper 2 cells
Tem: Effective Memory T-Cell
RT‒qPCR: Quantitative real-time polymerase chain reaction
IC50: Half maximal inhibitory concentration
