# Supplementary figures and images for: High FAAP24 expression reveals poor prognosis and an immunosuppressive microenvironment shaping in AML
Source: Cancer Cell Int. 2023 Jun 17;23:117. doi: 10.1186/s12935-023-02937-3 (PMC10276373; doi:10.1186/s12935-023-02937-3)

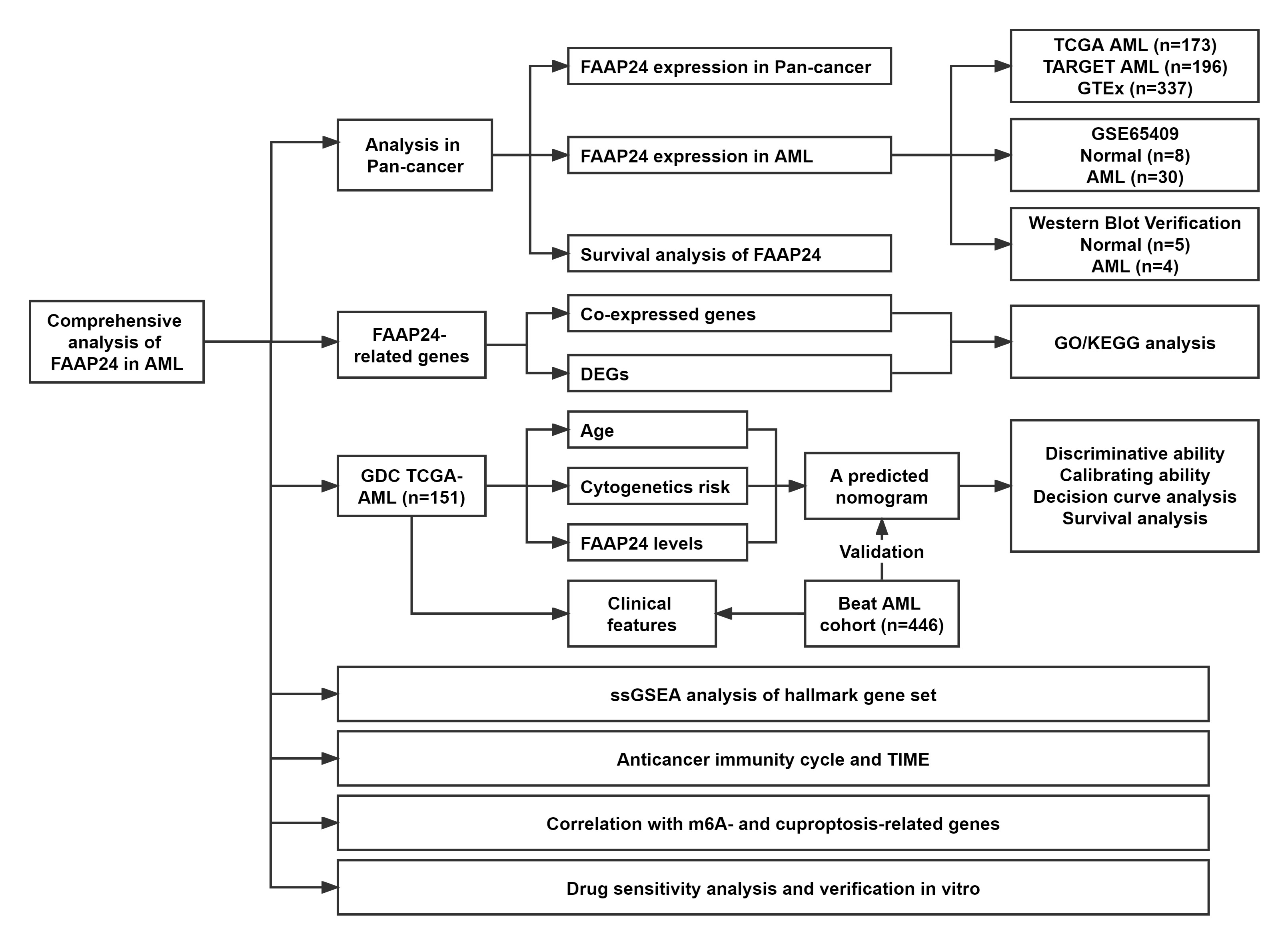

Supplement: Supplementary file 2 — Supplementary Material 2 [file 12935_2023_2937_MOESM2_ESM.jpg]
